# Acute Promyelocytic Leukemia in the Real World: Understanding Outcome Differences and How We Can Improve Them

**DOI:** 10.3390/cancers16234092

**Published:** 2024-12-06

**Authors:** Aram Bidikian, Jan Philipp Bewersdorf, Tariq Kewan, Maximilian Stahl, Amer M. Zeidan

**Affiliations:** 1 Department of Internal Medicine, Yale School of Medicine, Yale New Haven Hospital, New Haven, CT 06510, USA; 2 Section of Medical Oncology and Hematology, Department of Internal Medicine, Yale School of Medicine, Yale Comprehensive Cancer Center, New Haven, CT 06510, USA; 3 Department of Medical Oncology, Dana-Farber Cancer Institute, Boston, MA 02215, USA

**Keywords:** acute promyelocytic leukemia (APL), all-trans retinoic acid (ATRA), arsenic trioxide (ATO), clinical trials, survival, real-world outcomes, differentiation syndrome, clusters

## Abstract

The advent of all-trans retinoic acid (ATRA) and arsenic trioxide (ATO) has revolutionized the treatment of acute promyelocytic leukemia (APL), resulting in excellent rates of remission and long-term survival. However, real-world outcomes often fall short of those observed in clinical trials due to various factors related to patient demographics and clinical practices. This review examines APL treatment outcomes in real-world settings and highlights the phenomenon of APL clusters. Clinical trials frequently exclude older patients and individuals with significant comorbidities, yet these groups represent a substantial portion of patients in clinical practice. Early mortality remains high in real-world settings, compounded by delayed diagnosis and treatment initiation, as well as the inexperience of some community providers and limited resources of their centers in managing APL and its associated complications. High rates of disease and induction-related complications further exacerbate early mortality. Continuous education and collaboration between community healthcare centers and expert institutions are essential, and international partnerships between resource-limited settings and expert centers can improve global APL outcomes. Ongoing monitoring for measurable residual disease (MRD) recurrence and long-term treatment toxicity, coupled with comprehensive patient evaluations, and experienced management, can enhance long-term outcomes. The clustered incidence of APL, while frequently reported, remains poorly understood. Regular reporting of these clusters could provide valuable insights into disease pathology and aid in developing predictive models for APL incidence, which would guide future resource allocation.

## Simple Summary

The introduction of all-trans retinoic acid (ATRA) and arsenic trioxide (ATO) has greatly improved the treatment of acute promyelocytic leukemia (APL), leading to high remission and survival rates. However, real-world outcomes are worse than those seen in clinical trials, largely due to the exclusion of older patients and those with comorbidities from trials. Moreover, the results from clinical trials overlook patients who die of APL complications before registering for trials. Delays in diagnosis and treatment, along with the limited experience and resources of some community healthcare centers in managing APL and its complications contribute to higher early mortality rates. Ongoing education and collaboration between local healthcare providers and specialists are vital to improve APL outcomes. Regular monitoring for disease recurrence and management of treatment side effects are also important. Additionally, understanding the phenomenon of APL clusters—groups of cases over specific times or areas—can help improve resource allocation for better outcomes.

## 1. Introduction

Acute Promyelocytic Leukemia (APL) represents one of the greatest success stories of targeted therapy in cancer treatment. The advent of all-trans retinoic acid (ATRA) in the 1980s revolutionized the treatment of APL, showing high rates of complete remission as a single agent in early clinical trials [1,2,3]. However, these responses were often short-lived, necessitating the addition of anthracycline chemotherapy to the treatment regimen, especially among patients at higher risk of relapse, leading to longer durations of survival [4,5,6].

The landscape of APL treatment has experienced another paradigm shift with the discovery of arsenic trioxide (ATO) in the early 1990s which demonstrated high efficacy in the treatment of patients with APL who experienced relapse after ATRA therapy [7,8,9]. ATO’s success subsequently extended to first-line treatment settings, further improving patient outcomes [10,11,12,13]. Finally, two pivotal multicenter phase 3 clinical trials demonstrated the superiority of ATRA + ATO regimen over ATRA in combination with chemotherapy [14,15]. In these trials, the combination of ATRA + ATO, along with the later addition of gemtuzumab ozogamicin (GO) or idarubicin for higher-risk patients (defined as having a white blood cell count (WBC) greater than 10 × 10^9^/L at presentation), achieved complete remission (CR) rates of 93–100%, with induction mortality rates ranging from 0 to 4% and two-to-four-year overall survival (OS) rates of 93–99% [14,15,16,17]. Today, the ATRA + ATO combination is regarded as the gold standard for APL treatment, particularly for patients with lower- risk diseases, while the addition of GO or idarubicin is considered for those with higher-risk profiles [16]. Ongoing clinical trials are evaluating the role of oral arsenic formulations as well as retinoic acid agonist tamibarotene in the frontline and relapsed settings [18,19,20,21,22].

In this article, we explore the outcomes of APL treatment across multiple regions and settings outside of clinical trials. We discuss the associated challenges related to timely diagnosis, treatment, and prevention of early mortality. We also shed light on many efforts that positively impacted the outcomes in the real-world setting. Finally, we describe the clustered incidence of APL, a phenomenon that while frequently described, remains less well understood but has the potential to provide further insight into the risk factors of APL as well as future incidence patterns.

## 2. Overview of APL Epidemiology and Real-World Outcomes

APL represents between 5 and 20% of adult acute myeloid leukemia (AML) cases with an estimated annual incidence of 1 to 7.4 cases per 1,000,000 person–year [23,24,25]. The median age at diagnosis of APL is 47 years with slightly higher incidence among males compared with females [26]. Notably this median age is significantly younger than that of other AML subtypes [27]. Unlike most malignancies, APL’s incidence does not exhibit an exponential increase with age; instead, it remains relatively stable across various age groups. This pattern is reflective of the pathobiology of APL, which arises from a single rate-limiting translocation rather than an accumulation of mutations over time [27]. Therapy-related APL has been reported after exposure to topoisomerase inhibitors [28], and a single-center study in Japan has reported an increased incidence of APL among patients with non-small cell lung cancer treated with gefitinib [29]. In the US, APL incidence was found to be higher among Hispanics [30,31]. Additionally, a higher risk of APL incidence with increased body mass index (BMI) has also been reported [32,33].

Similarly to the outcomes with clinical trials, response and survival rates of APL in the real world had also improved over the past four decades, mirroring the improvements seen with the introduction of ATRA in the 1990s followed by the introduction of ATO in the 2000s [34,35]. Equally important in improving these outcomes were the substantial improvements in supportive care observed over the same period, as outcomes have improved with time even in the ATRA + ATO era [35,36]. These included advances in managing infections, differentiation syndrome, transfusion support as well as advanced management of associated bleeding diathesis [36].

Despite these improvements, outcomes in the real-world setting, whether reported from the review of national cancer registries or individual cancer center experiences, remain significantly inferior to those reported from clinical trials [35,37,38,39,40,41,42,43,44,45,46,47,48] (Table 1). While very few of these experiences report long-term survival outcomes (OS > 90% at 1 year) similar to those observed in clinical trials, the majority have inferior long-term OS rates ranging from 70 to 85% at 2–4 years. Strikingly, all these experiences report significantly higher early mortality rates (7–32%) when compared with <4% in the pivotal clinical trials (Table 1).

One common discrepancy between clinical trials and real-world outcomes is the patient demographics. Most clinical trials have very stringent inclusion criteria often excluding older patients and patients with higher-risk disease, significant comorbidities and lower performance status. Clinical trials of APL were not an exception. The median age of patients enrolled in clinical trials with ATRA + ATO was 45–47 years [14,15]. The APL0406 trial only included patients with low or intermediate-risk APL who had World Health Organization (WHO) performance scores of two or lower [14]. In the AML17 trial, patients with high-risk APL and worse performance status, while not formally excluded by the study’s inclusion criteria, only accounted for a minority of the enrolled patients (74% of patients had WBC of <10 × 10^9^/L and 71% had WHO performance score of zero) [15]. These trials also excluded patients with significant renal, hepatic or cardiac dysfunction [14,15]. Patient demographics in the real world are highly heterogeneous. The reported median age ranged between 30 and 54 years with patients ≥ 60 years of age constituting up to 36% of the population in certain databases [37]. The proportion of patients with high-risk APL was also significantly higher in the real-world setting, reaching up to 40–50% of patients [37,38,41,45]. Patients’ comorbidities and performance status, while not consistently reported in experiences from the real world, were also highly variable with one study reporting at least one comorbidity in 83% of the patients [39].

## 3. Early Mortality

**Figure 1:**
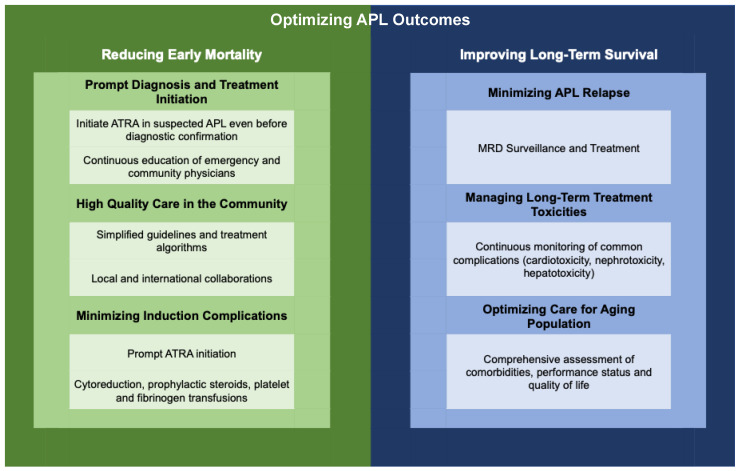
Barriers and Solutions to Optimizing Outcomes of Acute Promyelocytic Leukemia (APL) in the Real-World.

Early mortality usually is defined as death within 30 days of APL diagnosis, and remains a significant barrier to achieving optimal survival in APL. Most early mortality reportedly happens within the first 7 days of APL diagnosis (and even before diagnosis sometimes), while patients who survive beyond the first 7 days of APL treatment achieve up to 93% 5-year OS [40,44]. Early mortality has been recognized as a major risk of APL treatment in clinical trials; however, as reported above, early mortality rates in the real-world setting surpass those reported from clinical trials. Moreover, results from clinical trials likely often overlook patients who die of hemorrhagic complications before registering for trials [49]. The primary causes of early mortality in real-world cases mirror those seen in clinical studies, with intracranial bleeding accounting for over 50% of early deaths, followed by infections and complications related to induction therapy, such as differentiation syndrome and other major organ bleeding such as diffuse alveolar hemorrhage [39,44,48]. Several factors contribute to the increased rates of early mortality. These can be categorized into issues related to timely diagnosis and treatment initiation, the treatment setting and access to high-quality care, and the effective management of induction complications (Figure 1).

### 3.1. Diagnosis and Treatment Initiation

APL should remain high on the differential diagnosis of every patient presenting with acute/subacute pancytopenia or circulating blasts, particularly in the high-risk demographic groups, especially with the concomitant presence of clinical or laboratory findings of coagulopathy [50,51]. The APL evaluation should start with the emergent morphologic evaluation of peripheral blood smears for promyelocytes and Auer rods, which occasionally can be very rare precluding their observation before a marrow assessment is performed. However, the diagnosis of APL requires the detection of the pathognomonic t(15;17) or the *PML::RARA* fusion, which could be achieved through fluorescence in situ hybridization (FISH) or polymerase chain reaction (PCR)-based assays with slightly higher sensitivity of PCR compared with FISH [50,52,53]. Rarely, cryptic or atypical translocations, such as t(5;17), t(7;17), and t(14;17), might be missed by these assays, in which case testing with NGS could be beneficial [54,55,56,57]. While FISH and PCR assays have become more rapid and sensitive across the years, these assays may not be readily available in the community setting, which would necessitate sending out APL testing to expert laboratories, significantly prolonging the turnaround time for the results [58,59,60].

Prompt recognition of APL diagnosis and timely initiation of ATRA is of paramount importance to minimize bleeding and early APL mortality [61]. All current guidelines emphasize the importance of initiating ATRA in the setting of suspected APL even before diagnostic confirmation with FISH or PCR [62,63]. The use of NGS to confirm cryptic and uncommon translocations further extends the diagnostic turnaround time, underscoring the importance of initiating treatment before diagnostic confirmation. It is also highly recommended to start ATRA therapy in the emergency room for patients with suspected APL after (or in conjunction with) an urgent consultation with a hematologist [61]. ATRA could subsequently be safely discontinued if further testing rules out APL [61]. While early ATRA initiation has been successfully implemented in certain settings [64], a retrospective analysis revealed that even in highly experienced cancer centers, there was wide variation in the time of ATRA initiation, with only 31% of the patients receiving ATRA on the day of initial suspicion of APL [65]. Therefore, education on early recognition and treatment of APL should continue and should include emergency physicians, and community oncologists, in addition to academic hematologists [66]. Additionally, an exploratory analysis of 120 randomly selected hospitals in the U.S. found that only 31% had ATRA in stock [67]. The study identified three key barriers to availability: ATRA had not been recently requested by a physician, the inpatient pharmacist was unfamiliar with the drug, and the hospitals relied on affiliated cancer centers for supply [67]. These factors create additional obstacles to timely ATRA initiation especially if a timely transfer of the patient to a tertiary center is delayed, further contributing to the high early mortality rates.

### 3.2. Treatment Setting and Access to High-Quality Care

Reports from the real world consistently suggest that patients who are treated in academic or tertiary cancer centers experience better outcomes. Even among patients treated in academic centers, those treated in centers that care for a larger number of patients with acute leukemias per year experienced better outcomes than those treated in academic centers that treat fewer cases [37]. The better outcomes of treatment in academic centers could be secondary to a multitude of reasons, including the familiarity of oncologists with the most up-to-date treatment guidelines and the administration of treatment appropriate for the disease risk at presentation. A review of the Vizient clinical database revealed that while 92% of patients received treatment in academic teaching hospitals, only 80% of them received therapy that was aligned with NCCN guidelines based on their disease risk status. This discrepancy was linked to a higher risk of early mortality [37]. The findings suggest that treatment in specialized academic centers may be more beneficial than in community settings. However, referring patients to expert cancer centers can sometimes lead to delays in treatment initiation, which is a well-established risk factor for increased early mortality in APL [65]. Therefore, it is essential to explore more effective strategies to address these disparities in treatment access and quality.

One effective model was developed through the International Consortium on APL (IC-APL), an international collaborative network involving centers from Brazil, Chile, Mexico, and Uruguay, alongside experienced institutions from Europe and the USA [43]. This initiative aimed to adapt guidelines from expert centers to align with local resources. Additionally, it emphasized education and facilitated regular meetings between representatives from these centers. This resulted in significantly improved outcomes of APL treatment, including 50% reduction in early mortality rates when compared with historical outcomes [64]. Another successful co-management strategy was reported in a prospective multicenter trial by Jillella et al. [68]. This trial introduced a simplified algorithm aimed at the prevention, timely recognition, and treatment of early complications associated with APL. It emphasized dedicated education and frequent communication between community oncologists and APL experts, particularly during the critical first two weeks post-diagnosis, when most deaths are expected to occur. The trial demonstrated significantly improved short- and long-term treatment outcomes for patients with APL compared to historical data from the SEER database. Notably, the trial found no differences in outcomes between academic and community centers, highlighting the vital role of collaboration and education in the optimal management of APL in all contexts [68]. These positive results have prompted the ongoing national ECOG-ACRIN trial of a simplified care strategy to prevent early mortality in APL (NCT03253848) [69] (Figure 1).

Enhancing the capacity of community centers to provide advanced care is crucial, but it is not the only determinant of high-quality care for patients with APL. Equally important is addressing individuals’ ability to access such care, which is often hindered by various socioeconomic factors. Multiple studies have found a correlation between insurance status and survival outcomes: Uninsured individuals under 40 years and older patients with Medicaid or no health insurance often experience higher mortality rates [70,71]. Early mortality rates are also higher among individuals from rural areas and lower among patients presenting from low-poverty regions [72,73]. Therefore, ongoing efforts to improve access to care are essential to enhance outcomes for patients with APL. 

### 3.3. Induction Complications

Even with timely initiation of ATRA, patients with APL experience a variety of early treatment complications, including coagulopathy, differentiation syndrome, hyperleukocytosis, infections, and fluid overload [74,75].

The complex coagulopathy associated with APL often leads to intracerebral and pulmonary hemorrhages, which, to date, remain the most common causes of early mortality [62]. Coagulopathy in APL is primarily driven by fibrinolysis [76]. Promyelocytes in APL overexpress annexin II, which activates the tissue plasminogen activator (tPA), leading to enhanced fibrinolysis [77,78]. Annexin II is also highly expressed in the central nervous system, which may explain the increased risk of intracranial bleeding seen in APL [79]. While a consumptive process of clotting factors, as seen in DIC, is also present in APL, it is less likely to be the primary driver of coagulopathy, as evidenced by normal levels of antithrombin III and protein C and less pronounced abnormalities in aPTT [51,76,80]. Finally, the thrombocytopenia at the time of APL diagnosis further contributes to the elevated bleeding risk in the early stages of the disease [81].

It is estimated that 5% to 23% of patients with APL experience major bleeding during induction, with hemorrhagic death rates ranging between 3.7 and 8.4% [44,82,83,84]. Thrombotic events are also common during induction with ATRA + ATO with one study reporting 13(5%) of 248 patients experiencing major thrombotic events, including cerebral venous thrombosis and deep venous thrombosis of extremities [44]. Prompt initiation of ATRA has been observed to reduce the rate of mortality attributed to intracranial hemorrhages. Additional supportive measures for managing coagulopathy include daily monitoring of platelet counts and coagulation parameters and frequent transfusions to maintain fibrinogen > 100–150 mg/dL and platelets > 50,000 × 10^9^/L [62,74,85,86].

Differentiation syndrome (DS) has been reported to occur in up to 48% of patients treated with ATRA with severe cases reported in 12% of patients [87,88,89]. The use of cytoreductive therapy in high-risk patients and the incorporation of prophylactic steroids in APL treatment regimens has significantly reduced the rate of severe DS [14,90,91]. Nevertheless, DS still occurs in up to 20% of patients treated outside of clinical trials with one study reporting early mortality in 5 of 41 patients experiencing DS [44].

Leukocytosis, defined as WBC > 10 × 10^9^/L, is another feature of APL that can be present at diagnosis or develop during induction therapy. Leukocytosis can develop in up to 60% of patients with APL undergoing ATRA induction, with up to 12% of patients experiencing hyperleukocytosis (WBC > 50 × 10^9^/L) [92,93]. Hyperleukocytosis is typically considered a poor prognostic indicator, as it is associated with higher rates of early mortality and lower rates of both remission and long-term survival [92,93,94]. Management of hyperleukocytosis generally involves cytoreductive agents, such as hydroxyurea, along with prophylactic steroids to prevent severe DS [93,94]. Although leukapheresis has been performed in certain cases, it has not resulted in improved patient outcomes [92].

Infections are another significant complication in APL, occurring in 30–50% of patients undergoing ATRA induction and contributing to a higher risk of early mortality [41,48]. Fluid overload, often due to DS or large-volume transfusions, is another under-recognized complication that can lead to significant morbidity, including the need for ICU transfer and intubation in up to 10% of cases [95]. Therefore, early recognition of these complications and timely interventions are essential to reduce the disproportionately high rates of early mortality seen in real-world settings (Figure 1).

## 4. Long Term Survival

Long-term survival rates in APL have improved significantly over the years. An analysis of the SEER database revealed that patients diagnosed between 2002 and 2007 had a 3-year OS of 70%, compared to 55% for those diagnosed from 1992 to 1997 [42]. Similarly, patients diagnosed between 2010 and 2014 experienced a 4-year OS rate of 70%, in contrast to 66% for those diagnosed between 2005 and 2009. Importantly, the trend of improved OS remained significant even after excluding patients who experienced early mortality [35]. This improvement likely results from several advances in APL treatment, including the increased use of ATO in induction regimens, enhanced monitoring and intervention for measurable residual disease (MRD) recurrence, and advancements in supportive care.

In clinical trials, the ATRA + ATO combination was associated with a lower cumulative incidence of relapse (CIR) of 1% and higher rates of event-free survival (EFS) of 91–97% over a 2–4 year period, compared to ATRA combined with chemotherapy, which had a CIR of 6–18% and an EFS of 70–86% [14,15]. Similarly, real-world data showed that centers treating all patients with the ATRA + ATO regimen reported higher long-term EFS rates [41,44] than those that primarily used ATRA with anthracycline [38,45].

Clinical trials and retrospective studies have explored the role of a few biomarkers as prognostic indicators in APL. These studies revealed that *fms-like tyrosine kinase 3* internal tandem duplication (*FLT3*-ITD), higher expression of CD56, and lower expression of Preferentially Expressed Antigen of Melanoma (PRAME) are associated with shorter EFS and higher rates of APL relapse [96,97,98,99,100]. The use of these biomarkers in clinical practice remains limited, as they do not currently influence treatment decisions, but further research into these markers and others could potentially lead to changes in future treatment guidelines.

Although MRD recurrence in APL is rare with ATRA + ATO induction, it remains a strong predictor of morphologic relapse [101,102,103]. Prospective studies revealed that timely intervention with ATO has resulted in a significant reduction of overt relapse rates, suggesting the importance of MRD surveillance in APL [104,105]. MRD monitoring is performed using real-time quantitative PCR (RT-qPCR), a technique that, while effective, is also vulnerable to the limitations of diagnostic testing in resource-constrained settings as highlighted above. In the IC-APL collaborative model, MRD testing was conducted at designated national reference laboratories [64]. Although this approach may extend the turnaround time, it is acceptable in the context of MRD testing, as results are not as urgently required compared to initial diagnostic tests. In the IC-APL cohorts, MRD testing was performed every three months for two years. However, in resource-limited areas, it may be reasonable to limit relapse testing to the first year after induction, since relapses occurring after one year are quite rare [64,105].

As highlighted, treating patients in real-world settings often involves treating older individuals, who have poorer performance status, and present with more comorbidities than those typically enrolled in clinical trials. The poor survival rates in these patients are primarily due to higher early mortality, but a significant proportion also experience non-relapse mortality, suggesting the substantial role of their comorbidities and functional status in the poor long-term OS [106]. Therefore, managing these patients necessitates strong supportive measures and a heightened awareness of their comorbidities, which may be worsened by APL or its treatment [107,108,109]. ATO is associated with several long-term toxicities, including nephrotoxicity [110]. On the other hand, pre-existing kidney disease and reduced glomerular filtration rate (GFR) have been associated with increased ATO toxicity, requiring ATO dose adjustment [111]. In addition to nephrotoxicity, ATO is associated with cardiotoxicity and hepatotoxicity which require frequent ECG monitoring as well as kidney and liver function tests along with titration of other chronic medications that are metabolized by these organs on a case-by-case basis [16,110,112,113]. In a single-center experience, patients who were in complete remission for at least 3 years after treatment with ATRA + ATO+/−GO were found to have developed new comorbidities, the most common of which were hypertension (39%) and diabetes (26%), and they were using a median of three more chronic medications [114]. Long-term follow-up of patients who received anthracycline-based treatment for APL revealed 1–2% incidence of secondary myeloid neoplasms [115,116], whereas a subsequent multivariate analysis did not find a significant difference in the incidence of secondary myeloid malignancies among patients receiving anthracycline-based vs. anthracycline-free regimens [117]. Incidence of other secondary malignancies has been observed in patients with APL [35,117]; however, this rate is not significantly higher than that observed in the general population [35]. Therefore, it is highly likely that the increased occurrence of comorbidities and secondary cancers may be more related to the aging process and the chronic nature of APL rather than a direct association between APL or the treatment agents and secondary malignancies [35,112,113,114,118].

Finally, it is highly recommended that all older patients with APL undergo comprehensive geriatric assessment (GA), not only for its prognostic role but also to help tailor treatment decisions and guide supportive care [109,119]. GA will help tackle declines in physical function, identify cognitive and nutritional issues, and further elucidate the factors that influence the quality of life for older adults undergoing treatment for APL. The comprehensive evaluation of older adults with APL is essential for ensuring that their unique needs are addressed throughout the treatment process [109,120,121].

## 5. Clustered Incidence of APL

Over the past decades, the overall incidence of APL increased by 5.5% annually from 1992 to 2006 but remained stable since [73]. However, several studies noted “clusters” of APL presentations in certain geographic areas, where the incidence over specific periods has significantly surpassed the expected levels. The earliest report of clustered cases was from a hospital in South Africa, where nine children presented between the years of 1981 to 1985. This accounted for 7% of the hospital’s acute leukemia cases, while the estimated APL rate for that period was only 0–2%. Notably, seven of these nine children, presented from a well-defined geographical area in Eastern Cape [122]. In 2005, a hospital in the UK reported a cluster of three patients from the same geographical area who presented within four months (October 2002 to January 2003), significantly surpassing the expected incidence rate of one case per year at that center [123]. Recent temporal clusters have also been reported from Ireland, as well as Baltimore and Boston in the US, which significantly deviated from the local incidence patterns [124,125,126]. In Ireland, a cluster incidence of nine cases was reported in the first quarter of 2022, significantly surpassing the expected 2.125/quarter cases for that center. The demographics of these patients were similar to those in the general APL cohort and they came from various geographic locations [124]. A review of APL incidence patterns in Baltimore from 2000 to 2016 revealed that the overall incidence was lower than expected for the local population. However, the analysis identified twelve incidence clusters, each comprising four to five cases. These clusters were interspersed with prolonged periods during which there were no reported APL cases for several months. These clusters were not geographically confined and the demographics of these patients largely mirrored those of other APL patients [126]. In Boston, the incidence of APL between 2004 and 2013 aligned with the predicted rate of 1.52 cases per month for the metropolitan area. However, clustered presentations of five to six cases were observed in four separate months (May 2007, August 2008, March 2011, and April 2011). Notably, the median age of patients within these clusters was significantly higher than that of the general APL cohort (61 vs. 48 years). Additionally, the karyotypes of clustered patients showed a higher incidence of trisomy eight (22%) vs. (5%) in the general cohort. Other characteristics, including geographic background, were similar between the clustered patients and the broader APL population [125]. Of note, the temporal clusters did not correlate with local rates of upper respiratory viral infections [125].

Statistically, these clusters were unlikely to be attributed to chance alone, suggesting potential environmental risk factors that are yet to be defined [125,126]. Retrospective epidemiological studies have reported on associations between certain occupational and environmental exposures and increased risk of APL [127,128]. In a case–control study from Italy, there was a strong association between shoemaking and incidence of APL, as well as a moderate association between the incidence of APL and exposure to hair dyes and tuff (porous construction material containing elevated radon levels and gamma-emitting radionuclides) [127]. A later published exploratory case–case study from the GIMEMA database in Italy suggested a potential link between APL and electromagnetic fields, based on the increased incidence of APL among electrical workers and in the more industrialized regions of the country [128]; however, these associations were not consistently reproduced [129]. Further investigation into APL clusters could shed light on environmental risk factors and aid in developing disease incidence prediction models. This information would enable better allocation of local resources to facilitate timely diagnosis and treatment, which in turn, would lead to more effective disease management and improved patient outcomes.

## 6. Conclusions

Outcomes of APL treatment observed in the real world differ significantly from those reported in the pivotal clinical trials that established ATRA + ATO as the golden standard of APL treatment, often falling short of the high rates of complete remission and overall survival observed in these trials. This gap is partly due to the exclusion of older patients with poorer performance status and higher comorbidity burden from trials. Additionally, results from clinical trial results often overlook patients who die from hemorrhagic complications before enrollment.

The difference in outcomes are notable both in early mortality and long-term survival. Timely diagnosis of APL and ATRA initiation, even prior to confirmatory testing, are crucial in improving early mortality rates. This could be largely achieved through ongoing education for community physicians and emergency department staff. Collaboration between community cancer centers and expert treatment facilities, as demonstrated by successful models like the IC-APL, is also essential for enhancing early survival. Such partnerships elevate local care quality and adapt it to the limitations of locally available resources. Regular and standardized MRD monitoring enables early intervention in relapse cases, improving long-term survival rates. Furthermore, addressing long-term comorbidities—whether due to APL, its treatment, or aging—is vital for enhancing survival and maintaining quality of life among older adults with APL. Investigating APL clusters can provide insights into potential environmental or genetic risk factors, helping to predict future occurrences and proper resource allocation. All of this would help bridge the gap between real-world experiences and clinical trial results

## Figures and Tables

**Table 1:** Summary of APL outcomes from select studies in the real-world setting.

Study	N of Patients	Source	Median Age (≥60)	% High Risk	Early Mortality	CR, EFS and OS
Guru Murthy et al. [35]	2962	SEER (200–2014)	48 (20%)	NR	17–25%	4-year OS: 57–73%
Bewersdorf et al. [37]	1464	Vizient Clinical Database (USA: 04/2017–03/2020) ^^^^	53.5 (36%)	48%	14% *	NR
Dayama et al. [38]	34	Single center experience (India 2008–2012)	30 (NR)	41%	15%	4-year EFS: 65% 4-year OS: 75%
Ho et al. [39]	963	CCR and PPD (California:1999–2014) ^^	NR (NR)	NR	27% (7% in the first 7 days)	NR
Jacomo et al. [40]	134	12 centers in Brazil (2003–2006)	36 (NR)	37%	32% (25% in the first 14 days)	Median OS of 707 days
Kulkarni et al. [41]	123	Single center experience (India: 2006–2021)	34 (NR)	50%	6.5%	2-year EFS: 92% 2-year OS: 93%
Park et al. [42]	1400	SEER (USA: 1992–2007)	44 (36% **)	NR	17.3%	3-year OS improved from 54.6% (1992–1995) to 70.1% (2002–2007)
Rego et al. [43]	183	IC-APL (2005–2013)	34 (3.5%)	32%	15%	2-year DFS: 91% 2-year OS: 80%
Singh et al. [44]	256	Single center experience (India: 2006–2021)	32 (NR)	43%	12.1% (within the first 7 days)	2-year EFS: 81% 5-year EFS: 79% 2-year OS: 82% 5-year OS: 80%
Yedla et al. [45]	190	Single center experience (India 2006–2018)	33 (NR)	43%	21%	~3-year EFS: 69% ~3-year OS: 75%
Dhakal et al. [46]	7190	National Cancer Database (USA: 2004–2015) ^^^	50 (29%)	NR	12%	3-year OS: 75% 5-year OS: 71%
Lehmann et al. [47]	105	Swedish Adult Acute Leukemia Registry (1997–2006) ^	54 (NR)	NR	29% (one third of which happened within a day of diagnosis	16% of the patients who achieved CR relapsed at a median of 540 days 62% alive after a median follow-up of 6.4 years
Kim et al. [48]	313	5 academic center (South Korea 2000–2021)	50 (20% ***)	23%	13%	NR

^ Half were treated in university hospitals and the other half in non-university hospitals; ^^ Only 13% were treated in NCI-designated cancer center; ^^^ 53% were treated at an academic center; ^^^^ 92% were treated at academic centers; 64.8% were diagnosed in hospitals that treated ≥100 AML pts/year. 79.3% received NCCN guideline-concordant treatment for their risk category. * Defined as inpatient mortality or discharge to hospice; ** Age ≥ 54; *** Age ≥ 65; Abbreviations: CCR, California Cancer Registry; CR, Complete remission; DFS, Disease-free survival; EFS, Event-free survival; IC-APL, International Consortium on Acute Promyelocytic Leukemia; NR, Not reported; OS, Overall survival; PDD, Statewide Health Planning and Development Patient Discharge Database; SEER, Surveillance, Epidemiology, and End Results Program.
